# End-of-life situations in cardiology: a qualitative study of physicians' and nurses’ experience in a large university hospital

**DOI:** 10.1186/s12904-018-0366-5

**Published:** 2018-10-05

**Authors:** Fiona Ecarnot, Nicolas Meunier-Beillard, Marie-France Seronde, Romain Chopard, François Schiele, Jean-Pierre Quenot, Nicolas Meneveau

**Affiliations:** 1 Department of Cardiology, University Hospital Jean Minjoz, 3 Boulevard Fleming, 25000 Besançon, France; 2 EA3920, University of Burgundy Franche-Comté, 25000 Besançon, France; 3 Department of Intensive Care, François-Mitterrand University Hospital, 14, rue Paul Gaffarel, 21000 Dijon, France; 4 Department of Sociology, Centre Georges Chevrier UMR 7366 CNRS, University of Burgundy, 21000 Dijon, France; 5 Lipness Team, Inserm Research Center LNC-UMR1231 and LabExLipSTIC, University of Burgundy, 21000 Dijon, France; 6 Inserm CIC 1432, Clinical Epidemiology, University of Burgundy, 21000 Dijon, France

**Keywords:** End-of-life, Cardiology, Palliative care, Heart failure

## Abstract

**Background:**

Professional societies call for integration of end-of-life discussions early in the trajectory of heart failure, yet it remains unclear where current practices stand in relation to these recommendations. We sought to describe the perceptions and attitudes of caregivers in cardiology regarding end-of-life situations.

**Methods:**

We performed a qualitative study using semi-directive interviews in the cardiology department of a university teaching hospital in France. Physicians, nurses and nurses’ aides working full-time in the department at the time of the study were eligible. Participants were asked to describe how they experienced end-of-life situations. Interviews were recorded, transcribed and coded using thematic analysis to identify major and secondary themes.

**Results:**

All physicians (*N* = 16)(average age 43.5 ± 13 years), 16 nurses (average age 38.5 ± 7.6 years) and 5 nurses’ aides (average age 49 ± 7.8 years) participated. Interviews were held between 30 March and 17 July 2017. The main themes to emerge from the physicians’ discourse were the concept of cardiology being a very active discipline, and a very curative frame of mind was prevalent. Communication (with paramedical staff, patients and families) was deemed to be important. Advance directives were thought to be rare, and not especially useful. Nurses also reported communication as a major issue, but their form of communication is bounded by several factors (physicians’ prior discourse, legislation). They commonly engage in reconciling: between the approach (curative or palliative) and the reality of the treatment prescribed; performing curative interventions in patients they deem to be dying cases causes them distress. The emergency context prevents nurses from taking the time necessary to engage in end-of-life discussions. They engage in comfort-giving behaviors to maximize patient comfort.

**Conclusion:**

Current perceptions and practices vis-à-vis end-of-life situations in our department are individual, heterogeneous and not yet aligned with recommendations of professional societies.

## Background

Heart failure (HF) is a chronic, progressive disease that counts among the main causes of morbidity and mortality worldwide, affecting approximately 1–2% of the adult population in developed countries, and up to 10% of among those over 70 years of age [1]. Data from the EURObservational Research Programme reported mortality at 1 year of 17.4% in acute HF patients, and 7.2% in chronic stable HF [2]. Despite progress in medical and device therapy in recent years, cardiologists encounter a growing number of patients with advanced disease, often associated with multiple comorbidities. Deterioration of symptoms, and increasingly frequent hospitalizations for acute decompensation can herald worsening of the disease towards its ultimate stage and inevitably, death.

The guidelines issued by the European Society of Cardiology for the management of acute and chronic HF state that palliative care should be introduced early in the disease trajectory, and intensified as the disease progresses [1, 3]. Additionally, several recent reports have called for palliative care to be integrated into HF management [4–6]. Indeed, it has been shown that palliative care yields statistically and clinically significant improvements in patient’s quality of life, and is also associated with lower utilization of healthcare resources [7].

However, while these publications clearly identify the ideal strategies for implementing palliative care and discussing end-of-life goals for care with HF patients, it remains unclear where practices currently stand in relation to these goals, and how much ground we need to cover to achieve them.

To this end, we performed a qualitative study to describe and understand the perceptions and attitudes of physicians, nurses and nurses’ aides vis-à-vis end-of-life situations in the cardiology department of a university teaching hospital in France.

## Methods

We performed a single-centre, qualitative study using semi-directed interviews. All the medical and paramedical staff (physicians, nurses, nurses’ aides) working full-time in the Cardiology Department of the University Hospital of Besançon, France at the time of the study were invited to participate.

To be eligible, physicians had to be full-time, qualified staff cardiologists whose practice included delivery of care to patients in any of the units composing our Department, namely: (1) the acute cardiac care unit that admits cardiac emergencies directly (i.e. patients do not transit through the emergency room of the hospital); (2) a short-stay unit for scheduled invasive procedures; (3) a long-stay unit for hospital admissions for reasons other than scheduled procedures and for post Cardiac Care Unit care; and (4) an out-patient unit for ambulatory procedures. Clinical trainees who were not yet qualified MDs and visiting practitioners were excluded. Physicians were invited to participate by personal invitation.

To be eligible, nurses and nurses’ aides had to be full-time qualified staff, and could be from any unit, and on either day or night duty. Nurses were invited to participate through posters calling for volunteers in the common areas of each unit, plus oral information during staff meetings.

Interviews were performed between 30 March and 17 July 2017 by a qualitative researcher with experience in qualitative research, biostatistics and epidemiology (FE (female)) and by a sociologist with experience in clinical research and ICU care (NMB (male)). About the interview guide, participants were asked to describe how they experienced end-of-life situations in the Department. As with all qualitative interviews, the questions were open ended and intended as a prompt to get the respondent to talk about the aspects that were most important to them, and voiced in their own words. Interviews were performed in a private office within the Cardiology department. No other persons were present during the interviews.

All interviews were recorded in full and transcribed for later analysis. Data were encoded to guarantee the anonymity of the participants. Interviews were subsequently coded using thematic analysis as previously described elsewhere [8]. Briefly, interviews were coded independently by 2 of the coauthors (FE, NMB), the aim being to identify and categorize the different themes occurring in a cross-sectional manner across all interviews, (i.e. topics common to several individuals). Each theme is then considered as a meaningful independent unit of discursive language. The different themes that arise during the interviews are recorded, taking into account major themes (significant points that are of major importance and well developed by the participants) and secondary themes (less well developed by the participants). This first level of analysis was performed individually by each researcher, then meetings were held to harmonize and decide on the major and secondary themes to be retained, and their regrouping into subject categories. Differences in interpretation were resolved by discussion and consensus.

The study was approved by the Clinical Ethics Committee of the University Hospital of Besançon, France at its session on 8 February 2017. Informed consent was implied by the fact that all participants volunteered to be interviewed. Participants were made aware that quotes from their interviews may be used in scientific publications to substantiate the discussion. NVivo software was used to manage data (NVivo, version 11, QSR International, London UK).

## Results

All the physicians in the Department participated in the study (*N* = 16); the average age of physicians was 43.5 ± 13 years. In addition, 16 nurses (average age 38.5 ± 7.6 years) and 5 nurses’ aides (average age 49 ± 7.8 years) participated. Interviews were held between 30 March and 17 July 2017. Interviews lasted on average 38 ± 9 min for physicians, and 33 ± 10 min for nurses/nurses’ aides.

### Physicians’ perceptions

#### Active discipline

The first major theme to emerge from the discourse of the physicians was the view of cardiology as an active discipline, and as such, the physicians overall displayed a very “curative” frame of mind. Almost all the physicians cited the numerous technical, invasive or interventional procedures and devices, and the wide range of therapeutic options as an attribute of the discipline.

*“I was originally going to do sports medicine, but I did a rotation in Cardiology and I really appreciated the technicity of it” (MED010, ref4)*

*“It’s very “on-off”, and then there’s the technical side, we can do imaging, CT scanning, play around with computers, I like that kind of thing. And there’s the technical opportunities, we can put in valves, devices, it’s very gratifying. And I think the service rendered is excellent”. (MED008, ref3)*

As a result, they stated that there were always a lot of curative strategies that could be proposed for patients, almost like a “checklist” of options that they would implement one after another.

*“We’re always focused on care, there’s always positive proposals” (MED001, ref1)*

*“It’s very therapeutic and very active in terms of care, there are so many solutions, and a technical interventional side that I find interesting” (MED004, ref2)*

Several of the more senior physicians mentioned that this panoply of therapeutic options is a recent phenomenon, because with the outstanding progress achieved in recent decades in the care of heart failure, patients are now living longer, but they present more complex needs in the later stages of disease.

A further attribute of this very active nature of the discipline of Cardiology is the emergency context. Indeed, since cardiovascular emergencies are admitted directly to our Acute Cardiac Care Unit, these cases obviously take precedence, and accentuate the impression of an active, interventional discipline since resuscitation manoeuvers and emergency interventional procedures mobilize intense human and material resources in a short space of time. In this regard, several physicians emphasized that Cardiology does not allow the physician to time to ponder at leisure about end-of-life issues, but rather, calls for immediate action to prevent the patient from dying.

*“In cardiology, the end of life is generally quite sudden, and when it’s sudden and unexpected, we are very physically active and interventional, and we don’t really have the time to be asking ourselves all these questions” (MED003, ref1)*

*“Well, first we do the maximum at the start, to give ourselves a bit of time for reflection if there’s any doubt. We always start like that” (MED004, ref1)*

*“We’re very focused on the action when we discuss cases with colleagues, we talk more about what we’re going to do, or what works for the patient than about things that didn’t work but that we can do nothing about. For example, going to talk to a colleague about a difficult end of life situation, when the discussion will add nothing and the patient is probably already dead, well there’s no point really” (MED009, ref6)*

Indeed, several physicians found cardiology to be a gratifying discipline precisely because of the fact that a life-threatening event can be treated relatively rapidly, with a range of efficacious technical and medical strategies, often with good outcome for the patient.

*“It’s a bit like luxury plumbing. It’s logical, and easily explained. It’s very scientific”. (MED007, ref3)*

*“It’s a very active discipline. It’s a specialty that has reduced its mortality by 50% in 50 years, the only discipline to do that. It’s a specialty where we can do things, we’re pretty independent of the others” (MED009, ref6)*

#### Communication

A second prominent major theme in the discourse of the physicians was communication. Almost all physicians were in agreement that good communication is essential in promoting understanding and consensus, and that lack of communication can cause distress and incomprehension.

*“By the time you talk to the patient, the family, the team […] it can take all afternoon. But at least, everyone benefits. And then it’s so much easier after that in the unit, the family isn’t aggressive, nor is the patient” (MED002, ref9)*

*“I think it’s important, especially between the medical and paramedical staff, that there is some discussion and that everyone understands the rationale for the decision” (MED004, ref2)*

However, several physicians tended to equate “presence” with “availability for discussion”, whereby the simple fact of being present, in their minds, meant that for others, they were available for discussion, even of weighty matters such as end-of-life. Yet the circumstances they described did not appear to be compatible with meaningful and possibly time-consuming discussions of important life matters. In the view of the physicians, nurses have more time to spend with the patients, and therefore, could have a role to play in end-of-life discussions with patients.

*“I try to talk with the nurses a lot because they perceive more, they’re the ones who really take care of the patient” (MED005, ref6)*

Most physicians do not like to talk about end-of-life matters, and shirk the responsibility where possible, often with the excuse that they do not have the time for such discussions. Several senior physicians reported being called upon by younger and less experienced staff to handle “public relations”, such as dealing with families or announcing bad news, as their experience confers a certain poise and confidence. Those who said they do talk about end-of-life issues intimated that the discussions is not always intended to leave the choice open to the families or patient, but rather to explain things in a way that would bring the patient and family around to a certain realization, or in a way that would prompt them to adhere to the physician’s proposal. Some other physicians explicitly said that they prefer not to talk about death, as they fail to see the utility of dramatizing, and they feel that some patients are not receptive to discussions of this type.

*“Definitely, opening their eyes by telling them they have a serious and potentially lethal disease is one thing, but clearly, if they don’t want to hear it… and many people like to stick their head in the sand… being responsible is OK, but knowing all there is to know probably isn’t great either. I don’t know if it’s truly constructive for people to know everything.” (MED001, ref2)*

*“It’s true that the families – you really need to talk to them because you always think it’s clear in their minds, and that they understand that it will have to come to an end, but in fact no, not always. Or, they know but they can’t deal with it, they can’t accept it”. (MED007, ref2)*

*“…In my soul and conscience, I don’t know if I should tell someone “You’re going to die tonight”. Honestly, that’s not cool. Some people don’t want to know”. (MED006, ref2)*

#### Advance directives

Advance directives (AD) were addressed by several physicians, although in less depth that the previous two themes. The majority of patients admitted to our unit do not have AD.

*“I can’t remember ever having seen a patient who had advance directives” (MED004, ref1)*

*“It’s rare, it’s very rare” (MED007, ref1)*

*“It’s extremely rare in cardiology, extremely rare” (MED009, ref1)*

In any case, the physicians reported that they might not always take them into account, because they felt the patients were poorly qualified to know enough about the disease, the prognosis and the possible therapeutic options, to be able to make an informed decision.

*“Advance directives are not the be all and end all, because even if people have them, first of all we have to know about it, and it’s not always easy to find out especially if the person is admitted by the emergency services. We don’t have an exhaustive registry where people can register their AD. And even if they do have them, are they really qualified to say what level of care they want? Because that’s often the problem – the level of engagement. I don’t want go overboard, but the problem is that in the end, AD are often really vague and you’re back to square one, you still don’t know what the patient really wants” (MED013, ref2).*

*“It’s kind of hypocritical, we do ask about their preferences but […] I have to say that they shouldn’t really be taken into account. You have to let people have their say, and they express themselves quite crudely sometimes for someone who’s not all that sick, and right away, even just based on the age, they’ll tell you that they don’t want you to go overboard. That comes up regularly because people imagine we’re going to do all sorts of unimaginable things, even futile things. Above all, it underlines their anxiety, and their lack of knowledge about what’s possible and what isn’t. Of course when people say “I don’t want unreasonable obstinacy”, of course we listen, but in any case, we’ll do what has to be done, and it’s not the role of the son, daughter or husband to say “I prefer to condemn this patient”. Firstly, they know nothing about the medical situation, they don’t know the therapeutic possibilities, or the likelihood of recovery. And […] limiting care just because that’s what the next of kin wants, that makes no sense.” (MED003)*

They felt their professional knowledge might override the patient’s declarations, and the patient’s judgement could be clouded by severe disease.

*“A patient who tells us what they want or don’t want…. For us, that’s really rare. Because most people are here for serious cardiac problems that are difficult to treat, they have low cerebral blood flow so that doesn’t help facilitate comprehension or clarity of expression” (MED012, ref2)*

Most agreed nonetheless that AD cannot be a substitute for discussion with the patient and/or family, although it may represent too much of a burden on the family to give them final responsibility for end-of-life decisions concerning their loved-one.

#### Types of death

Another theme that was quite well developed was the distinction made by the physicians between two types of death in cardiology. Firstly, acute onset, sudden disease, which embodies the culmination of all the “active” characteristics of the discipline. In these emergency situations, the outcome is usually very rapid, and as such, death takes the need for end-of-life discussions out of the physicians’ hands.

*“In the acute cardiac care unit, unfortunately we don’t anticipate. We’re confronted with death, but it can’t be anticipated. It’s an emergency, and that’s all.” (MED010, ref2)*

*“I’d say [patients] die in one of two ways. There’s either the acute event – a massive infarction or resuscitated sudden death that turns out badly. That’s the acute situation. Or there’s the end-of-life in more chronic patients, especially patients with chronic heart failure whom we’ve been following for years, years and years…. They end up deteriorating, and then there comes a time when it’s over. Those patients are older, and those situations are completely different from the acute cases” (MED007, Ref1)*

An acute episode with resuscitation and emergency interventions requires immediate intensive action, and there is no time for end-of-life discussions.

*“For other acute events, besides infarction and its complications, it’s not up to us to decide. It’s black or white. An aortic dissection… well either you die or you don’t, and there’s no room for end-of-life discussions.” (MED012, ref2)*

In contrast, patients with chronic disease such as advanced heart failure, show a slower physical and cognitive decline, often with repeated hospital admissions at ever closer intervals. The visible gradual decline portends impending death and offers an opportunity, at each hospital admission, to address the end-of-life questions. In this case, informing the patient and family becomes the “technical” manoeuver.

### Nurses’ perceptions

#### Communication

As for the physicians, communication was a major theme for the nurses. However, their communication is bounded by several factors. First and foremost, the nurses’ communication is bounded by what the physician has already said to the patient.

*“What the doctor says is the divine word” (INF013)*

*“What does the family want, what does the patient want….. nobody asks us that very much. We’re spectators at the handover meeting in the morning, we’re not actors in the meeting, that’s for sure”. (INF014)*

Nurses state that they never take the initiative to address end-of-life issues with a patient if the physician has not already done so.

*“If the doctor hasn’t noted in the file that it’s now palliative care, then I wouldn’t speak to the family about it” (INF003)*

They also feel bounded by the legislation, in that they seek to relay a discourse that is in line with current legislation in France relating to patients’ rights at the end-of-life.

*“From a legal point of view, if there’s nothing noted in the medical file, then legally we’re obliged to perform resuscitation as usual” (INF014)*

Lastly, they are also bounded by the disease, insofar as chronically ill patients have more time to prepare for the end that those who are struck by sudden cardiac events.

In terms of communication with the medical team, the nurses serve as a relay between the patients and/or families and the physicians.

*“We’re often a sort of buffer for the family, we accompany them” (INF015)*

Indeed, their discourse suggests that they often try to convince the doctors to switch from a curative to a more palliative-oriented approach.

*“We find ourselves blocked by physicians who want to continue curative care, but without doing too much either…. We put a bit of pressure on the doctors, but we don’t really know where to position ourselves” (INF014)*

In this regard, they often perform informal debriefing among themselves to discuss end-of-life issues, especially when the physicians’ instructions are not in line with their perception of what should be done.

#### Reconciling

The second major theme to emerge from the paramedical staff was that they are permanently engaged in various types of reconciling. First, they try to reconcile the incoherence between the approach (curative or palliative) and the reality of the treatment prescribed, which is beyond their control. For example, being instructed to continue blood samples and monitoring for a patient whom they clearly consider to be a “palliative case” represents an incoherence that they have to reckon with, and this can cause distress for nurses.

*“The problem is the viewpoint – Because one minute, the patient is at the end of life, say for example the weekend; then on Monday, he’s resuscitated and you have to do everything all over again, take more workups […] and then in the end, the doctor says, “Anyway, there’s nothing more we can do”. So if there’s nothing more we can do, why do you keep asking me to take blood every day?” (INF013)*

*“Sometimes you’re caught between two situations, we continue curative care and then it’s half curative, half palliative. You’re giving dobutamine and then giving morphine at the same time. After a while, you just think, it’s not coherent!” (INF014)*

*“It’s difficult when you’re not being heard or respected as a nurse, when you’re sounding alarm bells and they [the physicians] just respond “no, no, that’s not how it is….”” (INF013)*

*“Sometimes one doctor does one thing, then the next day it’s another doctor who does rounds and they change everything. We really need everyone to be in agreement on the management”. (INF014)*

Second, they have to reconcile the time constraints of the emergency context with the time-consuming nature of end-of-life discussions; while they might be willing to take the time necessary to talk about the end-of-life with certain patients, the reality is that if an emergency is admitted in the meantime, they must deal with the emergency first.

*“It’s not always possible [to optimize the end of life], sometimes it’s too late, sometimes it’s too difficult, and we have to take account of so many external parameters” (INF008)*

*“You have to take care of the living first”. (INF003)*

*“It’s true that a 90-year old who’s dying slowly, and a young 30-year old with an infarction … so who do you take care of first? We’re not supposed to be practising medicine like on the battlefield where you prioritise some patients over others. We’re not supposed to have to choose… we’d like to be able to give the same amount of time to everyone, but…..”*

Similarly, for nurses not working in the acute cardiac unit, the workload is such that the ordinary tasks that need to be completed take precedence over end-of-life discussions. Third, nurses often serve as a relay to reconcile the naivety of younger colleagues or young physicians (i.e. believing you can save everyone) with the reality of life (first experience of losing a patient, first time seeing a cadaver etc).

*“Maybe it’s harder for some physicians to admit that a youngster of 21 needs palliative care, rather than a little old lady…. They get it hard enough when it’s old people, so for a youngster…. I’m going to wait and see how that turns out” (INF015)*

*“[Younger colleagues are] not always prepared because death is always abstract as long as you haven’t experienced a situation like that, and as long as you haven’t actually had a dead person in the bed in front of you” (INF008)*

*“The paramedical staff accept more easily to let a patient go, whereas the doctors always take a curative approach at all costs”. (INF013)*

Finally, another relay-role is to reconcile the patients’ lack of knowledge, interest or awareness about their prognosis, with the reality of the clinical situation, especially when this reality is impending death.

#### Comfort-giving

The overriding concern for the nurses is to ensure the patient’s comfort at all times.

*“Really, the aim is just to relieve the patient as much as possible” (INF014)*

They mention such actions as being flexible about visiting hours for families of dying patients, chatting with the patient, “just being there”.

*“We welcome the family, we accompany them, we try to make the patient presentable for the family, we ask them if the need anything, if they need to talk we try to make ourselves available […] we try to respect everyone’s rituals” (INF015)*

*“I even cried in the room with the family once when the patient died” (INF008)*

All of the nurses’ actions are basically motivated by the fundamental desire to maximize the patient’s comfort, even if this involves coaxing the physician towards a palliative approach so as to relieve the suffering they perceive the patient to be experiencing.

### Nurses’ aides perceptions

#### Communication

In line with the perceptions reported by nurses, communication was a major theme for the nurses’ aides. Since they spend significantly more time with the patients, performing intimate tasks such as bathing, they have the time and opportunity to establish a more personal and intimate connection with the patients. In this regard, they often find themselves privy to confidences from the patient. When appropriate, they understand that it is important for the purposes of communication that they (the nurses’ aides) forward this information to the nurses (or physicians) to ensure that it is taken into account.

*“Yes, sure, we’re there to listen. But we’re not the ones who do it, we pass on the info to our nurse or doctor and then it’s taken care of, it’s group work. So yeah… everybody is … well, bound together, everyone does what they can” (Nurse’s aide 001)*

“*the patients… the families, they talk to us. Quite a bit actually.” (Nurse’s aide 002)*

They also reported that communication with the Palliative Care Department of our hospital is now more frequent than before, and this is helpful for managing difficult end-of-life situations:

*“I think we’ve really made progress [as regards] the pain… management of pain. And now we also call in palliative care” (Nurse’s aide 001)*

*“Management in terms of palliative care is better, they call them more and more often now. That’s a big step forward, I think. Because they help us a lot. They really help us a lot.” (Nurse’s aide 002)*

#### Comfort-giving

Again, similar to the nurses, the nurses’ aides play an important role in comfort giving for end-of-life patients. This role is mainly fulfilled through simply being there for the patient, and for the families.

*“We try, at the same time we try to stay discrete, but we try to be close to the family, let them know we’re there for them, if they need to talk. I think that helps” (Nurse’s aide 005)*

*“We are there to listen to our patients. Not so long ago, we had this little old lady who wanted champagne. So we opened a bottle and gave her a glass and… well… She was so delighted. […] She passed away really relieved, she was at peace”. (Nurse’s aide 003)*

*“Generally, we try to accompany them as best we can, we don’t let them die alone. If we can, we hold their hand. We try to make sure at least that they’re not alone”. (Nurse’s aide 003)*

*“[we try to make sure] that someone is always there for them.” (Nurse’s aide 004)*

## Discussion

This study reveals several interesting findings regarding the perceptions that physicians and nurses have about end-of-life issues in a large, university hospital cardiology department. Firstly, physicians present an overwhelmingly curative frame of mind, and view the discipline of cardiology as a very active one, with a large therapeutic armamentarium. Their aim is to provide curative care at all costs. Second, nurses, on the other hand, clearly take a different but complementary approach. They have a less invasive attitude, and are more inclined to acknowledge that patients are at the end-of-life and should be labelled as palliative care. The nurses seem eager for there to be some guidance, or official consensus about when a patient should be considered to be in palliative care, and how this decision should be materialized, since the changing attitudes of different physicians who successively take care of the patient can cause incomprehension and as a result, distress among the nurses. Overall, our findings show that practices are highly individual, differ between medical and paramedical staff, and in any case, are not at all standardized.

Practices are currently far from the recommendations of professional societies regarding advance care planning and end-of-life discussions [1, 3], although few concrete indicators exist as to how this should be materialized in the practice of cardiology. Indeed, in their review of existing quality indicators in national Swedish policy documents relevant to palliative and end of life care, Lind et al. reported that no indicators relevant for palliative or end of life care were present in guidelines in the field of cardiology [9]. The Australian National Heart Foundation guidelines for multidisciplinary care for heart failure patients are useful in this regard, as they lay down clear directions with checklists for practical use in implementing their recommendations for best practice [10, 11]. Similarly, there has been some discussion recently regarding the triggers that should prompt physicians to initiate end-of-life conversations or advance care planning. For example, Denvir et al. reported from an interview study with patients and carers that an estimated 1-year mortality risk of 20% or higher should prompt physicians to initiate end-of-life planning [12, 13].

The active and curative attitude of the physicians reveals what they consider to be the boundaries of the discipline of cardiology. Indeed, they describe a therapeutic approach akin to a conveyor belt of care: once the patient is admitted and steps onto that conveyor belt, they are moved along as the physician proposes one therapy, intervention or device after another, until all possibilities have been exhausted. Indeed, in a secondary analysis of qualitative data collected through the Leadership Saves Lives initiative, Flieger et al. described this phenomenon as the “power of momentum”, whereby once the patient presents with an acute need, that increases the likelihood of intervention [14]. With the outstanding progress in medical and device therapy in recent years, there is now a very large choice of therapies available, and thus, for a long time during the course of disease, physicians can offer a new therapy every time the previous one has failed. In a recent qualitative study investigating the reasons why doctors provide futile treatment at the end of life, Wilmott et al. reported that one of the main drivers of futile end-of-life care was the characteristic of treating doctors of being oriented towards curative treatment (“the therapeutic imperative”), and the desire to satisfy the patient, the family and the medical professionals themselves that “everything possible had been done”. [15]. The result is that the physician is always in a state of expectation, waiting to see the effects of the most recent therapy – and very often, it may work, and the patient may recover. But the successive proposals also serve to postpone the moment when it will become necessary to talk about the end-of-life. Indeed, for a variety of reasons, palliative care is not often proposed to cardiac patients, or may be proposed too late in the trajectory of disease, thereby denying cardiac patients access to end-of-life care [16, 17].

For the physician, once that patient has reached the end of the “cardiology” conveyor belt, and the only remaining option appears to be inevitable death, then most physicians believe that it is someone else’s job to take over the patient’s management beyond that point, i.e. from there until death (e.g. the nurses, or the intensive care unit, or the palliative care department [17]). In a survey of clinical attitudes and self-reported practices regarding end-of-life care in heart failure, Dunlay et al. reported that among 95 clinicians interviewed, many reported discussing end-of-life wishes when the patient’s health status worsened, and that the most common reasons for referral to palliative care were that the patient had no other heart failure therapeutic options [16].

The American sociologist Everett Cherrington Hughes, in his work entitled “Men and Their Work”, describes how professionals pose the limits of their professions, and stipulate the division of labor within this context [18]. Our cardiologists appear to consider death to be beyond the boundaries of their profession, and not to be part of their role in the division of labor. As Hughes also stated from sociological observations, “…the delegation of dirty work to someone else is common among humans” [19], and this appears to be a basic human behavior at play here. The physicians of our Department believe their role is to cure and to offer therapeutic solutions, and they would gladly have someone else take care of the patients who are dying and can no longer be saved, and who are, as such, no longer candidates for the highly active and interventional opportunities the cardiologist can offer. This finding is in line with the findings of the qualitative study by Flieger et al., where respondents indicated that a lot of cardiology professionals are afraid of palliative care, and that cardiologists “don’t believe in it” [14]. The authors relate this rejection of palliative care to the tendency towards intervention among cardiologists.

With all the therapeutic solutions at their disposal, cardiologists are not yet accustomed to thinking about when these therapies might reasonably be de-escalated or scaled back. Indeed, the “trained to treat” attitude described by Willmott et al. in their sample of 96 physicians from various disciplines underpins the perception of many physicians that death is a failure, leading to a reluctance to acknowledge the role of palliative care [15, 20]. Physicians in our study cited lack of time as a reason for not engaging in end-of-life discussions. In a recent telephone survey of physicians in the United States, Fulmer et al. reported that the number one barrier to advance care planning conversations, cited by two-thirds of respondents, was lack of time [21]. In the case of the respondents in our study, this may just be an excuse, since most people are naturally averse to talking about death. Instead of saying they do not have the time, it might be more accurate to say that they do not take the time. It has previously been reported that discomfort with end-of-life conversations leads physicians to avoid addressing the topic with the patient [15], while overall, 30% (28 of 94) of clinicians reported “low” or “very low” confidence in initiating EOL or hospice discussions in the survey by Dunlay et al. [16].

At this juncture, the nurses’ role takes on its full importance, as they take over the care of the dying patients. Indeed, they are often instrumental in bringing the patient to this point, in that they coax physicians to stop curative therapies, or invasive monitoring, and increase comfort care, with a view to leaving the patient to die in peace. When there is incomprehension about the physician’s intent, or prescriptions for curative interventions for a patient whom the nurse feels to be dying, then this creates distress among the nurses. This is coherent with previous reports of high moral distress among nurses in critical care units, particularly due to futile care or physician-related factors, such as having to assist a physician who the nurse feels is incompetent [22]. The incomprehension can be due to lack of communication, as the physicians always believe they have a sound scientific rationale for their prescriptions, but the nurses may have a different perception from having spent more time with the patient, and often also with the family. In a prospective opinion survey of critical care providers, Frick et al. reported that nurses gave more pessimistic judgments and considered withdrawal more often in dying patients than doctors did [23]. These authors also relate this finding to the fact that, as observed in our study, nurses spend more time with the patients than the physicians, often accompanying them through emotionally charged circumstances. For this reason, they may be more aware of the suffering of the patients, and more inclined to admit treatment failure [23]. Families and patients often confide things in nurses that they won’t say to the physician. The role of the nurses in communicating the wishes and values of the patients and their families when discussing individual cases is thus vital, and the information they can provide is complementary to the physician’s predominantly medical evaluation. This is coherent with a previous report of a grounded theory study among nurses in intensive care units in nine countries, which reported that although nurses do not make the final end-of-life decisions, they engage in the core practices of consensus seeking, which involves coaxing, relaying information and giving voice to patients’ feelings, and emotional holding, which involves comfort-giving [24]. Although the nurses seem willing to take on the role of having end-of-life discussions with the patients, they do feel restricted in the scope of their conversations with patients by what the physician has already told the patient. In this regard, in line with efforts to involve all healthcare professionals in improving end-of-life management across the spectrum of the healthcare pathway, it is essential that nurses be involved in the discussions regarding end-of-life decisions, and they may also be involved in the communication with the patient about end-of-life matters. According to a survey performed by Aleksova among cardiologists, cardiology trainees and cardiology nurses providing care for heart failure patients, nurses were more willing than physicians to initiate and engage in end-of-life discussions with patients [25]. In this same study, among non-physician clinicians, advanced practice nurses were deemed to be most acceptable to be involved in decision-making relating to goals of care [25]. However, it is important that the physicians do not shirk responsibility by having nurses play a role that the patient traditionally expects the physician to play. This could be perceived by the patient as being abandoned by the physician.

Advance care planning clearly remains underutilized in our region. Advance care planning is a dynamic process that aims to encourage and empower the patient to discuss their disease course, prognosis and likely outcome with their family and with their physician, especially the healthcare trajectory they wish to follow [26]. The patient can consign his/her preferences and desires for end-of-life care in written form, such as AD, in case he/she subsequently becomes decisionally incapacitated. Advance care planning aims to ensure that patients receive care that is in accordance with their wishes, particularly at the end-of-life, and also helps to reduce unwarranted interventions [26, 27]. Despite evidence that advance care planning positively impacts on end-of-life care [26, 28], a range of barriers remain to effective and systematic implementation of such services in clinical practice [28, 29]. In France, despite a generally positive attitude towards AD among the general public, reportedly only 2.5% of patients have actually drafted AD [30, 31]. In a recent review of AD from hematology departments, Trarieux-Signol [31] et al. describe the current French legislation in detail, noting in particular that AD are binding, except when “the content of the AD appears to be manifestly inappropriate or inconsistent with the patient’s medical condition”. This is coherent with the findings of our study, where physicians said that while they will enquire if AD exist and take note of what they contain, they might actually pursue a different course of action if they judge the content of the AD to be “off the mark” or blatantly irrelevant to the patient’s condition. This echoes the findings of Aleksova et al., who reported that family members’ difficulty accepting the poor prognosis of a loved one, and the patient’s own difficulty understanding their prognosis and therapeutic options represent significant barriers to goals-of-care conversations [25].

This attitude, whereby the physician and family and/or other bodies, such as the state, may override the individual’s choice in the name of a greater good, is more common in European countries, and in clear opposition with the American attitude whereby the patient’s autonomy prevails above all [32]. Indeed, in their investigation of the challenges and opportunities for engaging palliative care after myocardial infarction, Flieger et al. reported the difficulty of delivering care that is aligned with the patients’ preferences when there are limited opportunities to ascertain what these preferences may be, once treatment is under way [14]. In this context of different societal attitudes to such key questions as patient autonomy, it is likely that culturally-specific models of end-of-life care are warranted to ensure that practices are in harmony with the prevailing principles of healthcare delivery and perceptions of the doctor-patient relationship. Similarly, it may be difficult to implement “one size fits all” recommendations for end-of-life care in the presence of such starkly different world views. Efforts to correct misperceptions about the meaning of palliative care (equating it with end-of-life care and pending death) may be warranted. Also, a multidisciplinary team approach with coordinated collaboration between healthcare professionals could help to ease transitions between healthcare providers, make appropriate care and symptom relief available in a timely manner, and achieve the desired outcome of a peaceful death in the location of the patient’s choice [10].

This study suffers from several limitations that deserve to be underlined. Firstly, it is a single-centre study and therefore, may not be generalizable to the whole population of physicians and nurses/nurses’ aides working in cardiology. However, the volume and type of activity, as well as the profile of the physicians and nurses is similar to that observed in other, similar sized university hospital cardiology departments, so it is plausible that while practices may vary according to local culture, general attitudes among this professional group are likely similar elsewhere. Although our results are not wholly unexpected, they nonetheless open avenues for further, multicenter studies that would help inform about practices in other, similar sized institutions. Secondly, other hospital personnel likely to be included in end-of-life discussions, such as intensive care unit physicians, palliative care physicians, social workers and psychologists, were not included in this study. Thirdly, we cannot rule out the possibility that previous memorable experiences of participants, either positive or negative, and in either their personal or their professional lives, may have influenced the discourse substantially. Fourth, a wealth of other topics were raised by both groups of professionals that we are unable to address here for lack of space. These points warrant further exploration and analysis. Finally, it would be interesting to complement this analysis by interviewing families who have experienced the end-of-life of a loved one in our Department, in order to understand the impact of the caregivers’ attitudes on the families.

## Conclusion

In conclusion, this qualitative study in the cardiology department of a large university teaching hospital shows that physicians and nurses have different, yet complementary attitudes to end-of-life issues. Both approaches must align towards a common goal, namely integrating discussions about end-of-life goals of care across the disease spectrum where possible, with a view to improving communication and maximizing patient comfort at the end-of-life. Practices in our Department are heterogeneous, and fall short of the objectives outlined by professional societies in this regard, particularly in patients with advanced heart failure. There is a compelling need for a minimum of training in palliative care skills among cardiologists. Improved training would help provide clinicians with the ability to anticipate end-of-life discussions, and improve communication skills. A change in the paradigm of what the discipline of cardiology encompasses is also warranted, in order to integrate palliative care in a systematic and standardized manner.
